# Do Open Fracture Classification Systems Predict Functional Outcomes in a Low-Income Country? A Prospective Study of 287 People with Open Tibia Fractures in Malawi

**DOI:** 10.2106/JBJS.OA.25.00090

**Published:** 2025-09-11

**Authors:** Alexander Thomas Schade, Vincent Mkochi, Nohakhelha Nyamulani, Maureen Sabawo, Kaweme Mwafulirwa, Chikumbutso Clara Mpanga, Leonard Banza Ngoie, Andrew John Metcalfe, William Jim Harrison, Peter MacPherson

**Affiliations:** 1 Malawi-Liverpool-Wellcome Trust, Blantyre, Malawi; 2 University Hospitals Coventry and Warwickshire, Coventry, United Kingdom; 3 Kamuzu Central Hospital, Lilongwe, Malawi; 4 Queen Elizabeth Central Hospital, Blantyre, Malawi; 5 Mzuzu Central Hospital, Mzuzu, Malawi; 6 University of Warwick Medical School, Coventry, United Kingdom; 7 Countess of Chester NHS Foundation Trust, Chester, United Kingdom; 8 AO Alliance, Davos, Switzerland; 9 University of Glasgow, Glasgow, United Kingdom

## Abstract

**Background:**

Open fractures are common and severe injuries that are associated with poor functional outcomes and quality of life, and high societal costs. Several classifications systems have been developed to characterize these injuries, predict prognosis and plan treatment. We aimed to assess the agreement between open fracture classification and patient-reported function, fracture-related infection, and amputation.

**Methods:**

In this prospective cohort study, we enrolled adults with open tibia fractures in 6 hospitals across Malawi. Radiographs and clinical photographs were classified according to the Gustilo-Anderson, Orthopaedic Trauma Society (OTS), Muller and Tscherne classification by an orthopaedic surgeon. Participants' function (using the Short Musculoskeletal Assessment Score), and risk of fracture-related infection and amputation were assessed by face-to-face interviews at 6 weeks, 3 months, 6 months, and 1 year postinjury. The Kendall rank correlation coefficient with 95% bootstrapped confidence intervals investigated correlation between fracture classifications and patient outcomes.

**Results:**

Two hundred eighty-seven participants were recruited with 252 of 287 photographs (88%) and 274 of 287 radiographs (95%) available for review. The Kendall correlation with function score 1 year after injury was 0.34 for OTS classification, 0.18 for Gustilo, 0.17 for Tscherne, and -0.02 for Muller. For correlation with fracture-related-infection at 1 year, this was 0.34 for the Orthopaedic Trauma Society, 0.31 for Gustilo, 0.24 for Tscherne, −0.02 for Muller. For amputation, correlation was 0.39 for OTS, 0.24 for Gustilo, 0.24 for Tscherne, and 0.12 for Muller.

**Discussions:**

Most open fracture classification systems had negligible or weak correlation with subsequent patient function, fracture-related infection, or amputation in Malawi. Treatment factors and other confounders may have a greater influence on outcomes, but classification systems must still account for this variability to remain useful. More research in low or middle income countries needs to be conducted to develop appropriate and relevant open fracture classifications to improve and standardize the management of open fractures.

**Level of Evidence:**

Level II. See Instructions for Authors for a complete description of levels of evidence.

## Introduction

The vast majority of injuries (90%) occurs in low- and middle-income countries (LMICs), but very little orthopaedic research is conducted in these countries^1^. Open tibia fractures are severe and common injuries in LMICs, mostly due to the rapid increase in road traffic motorcycle incidents, which frequently result in mortality, severity morbidity, and high societal costs^2^.

Classification systems can help guide clinicians to characterize fracture severity, support prognostication and plan treatment, and help researchers to uniformly report, document, and compare clinical and epidemiological data^3^. Classifications need to be valid and reliable; however, it is estimated that 70% of trauma and orthopaedic classifications have never been independently validated or assessed for intraobserver and interobserver agreement^4^. Some of the commonly used classifications for open fractures include the Gustilo-Anderson classification^5^ and the Orthopaedic Trauma Society^6^. In addition, wound classifications such as AO Tscherne^7^ and fracture severity classifications such as AO Müller^8^ can provide further insights, particularly when examining their correlation with outcomes. These classifications have been shown to have good correlation with patient outcomes in high-income countries^5,6,9^.

The Gustilo-Anderson classification, developed in 1976, correlates with infection and amputation risk in HIC and remains the most commonly reported system for open fractures, particularly in low-income settings^5^. The Orthopaedic Trauma Society (OTS) classification correlates with patient functional outcomes in large UK cohort studies and report improved intraobserver and interobserver agreement compared with other classifications such as the Gustilo classification^6^. The Tscherne system, which grades wound severity and tissue damage, is less commonly used in outcome studies^7^. The Müller classification describes fracture location and pattern but is not specific to open fractures^8^. Despite their widespread use, no single classification has demonstrated clear superiority in predicting key patient outcomes such as infection, amputation, or functional recovery^10,11^.

Malawi is a landlocked low-income country in sub-Saharan Africa with a population of approximately 21 million people in 2023. Data on the burden of injury in Malawi remain limited, but the World Health Organization estimates that road injuries were among the top 5 causes of death and disability between 2010 and 2020^12^. The national government–funded health service has 3 levels of care with the majority of fractures being managed nonoperatively and care delivered by orthopaedic clinical officers (nonphysicians) in district and tertiary hospitals^13^. Efforts have been made to standardize and improve open fracture care based on the severity of injury^14^ and to centralize treatment of more severe Gustilo III injuries to tertiary hospitals, as this improves patient function in the year following injury^2^. Therefore, a better understanding of the open fracture classification could improve referral and management of patients with open fractures and ultimately their outcomes^15^.

There are major issues with the current classification systems used to grade severity of open fractures in low-income countries. Although historical classification systems, such as Gustilo, were created for purposes of directing treatment and making predictions for complications such as infection, it is not known whether those same classifications have any relationship with patient-important outcome measures such as function, pain, and return to everyday living. Qualitative studies in recent years have found that for the patient, a great deal of importance is placed on independence regained, return to employment, and limiting long-term disability^16^. It is currently unknown if these classifications can predict complications or patient outcomes in a different setting such as a low-income country^6^. The aim of this study was therefore to determine whether the current classifications for open fractures are correlated to complications and patient-reported outcomes in a low income country (LIC).

## Methods

### Study Design and Participants

This study was nested in a prospective, multicenter cohort study conducted in 6 hospitals in Malawi: 2 tertiary hospitals (Queen Elizabeth Central Hospital and Kamuzu Central Hospital) and 4 district hospitals (Dedza District Hospital, Ntcheu District Hospital, Balaka District Hospital, and Machinga District Hospital). The study was approved by the College of Medicine Research and Ethics Committee in Malawi and the Liverpool School of Tropical Medicine in the United Kingdom. Written informed consent was obtained from all patients in the study. The study protocol has been published previously^17^. The study is reported in accordance with Strengthening the Reporting of Observational Studies in Epidemiology (STROBE) guidelines.

Potential participants were systematically screened for study inclusion by health workers (orthopaedic clinical officers or orthopaedic surgeons in the 6 hospitals) who had received study-specific training at workshops. Eligible participants were adult patients (aged ≥18 years) who presented to hospital emergency departments with an open tibia shaft fracture (as per The Arbeitsgemeinschaft für Osteosynthesefrage Foundation/Orthopaedic Trauma Association class 42^8^) between February 12, 2021, and March 14, 2022. Patients with additional injuries beyond the open tibial fracture were included. We excluded people who were unable to consent to study participation or were unable to complete patient-reported outcome questionnaires.

### Procedures

Available radiographs (anterior-posterior and lateral) and clinical photographs were reviewed and classified by a single orthopaedic surgeon (Table I). If photographs or radiographs were missing, documented classification by the treating clinician was noted. Borderline or unclear radiographs and clinical photographs were discussed with senior orthopaedic surgeon (W.J.H.).

**TABLE I:** Classification of Open Fractures

Classification	Description
Gustilo (6)	Type I: skin wound less than 1 cm, clean, simple fracture pattern
	Type II: skin wound more than 1 cm, soft tissue damage not extensive, no flaps or avulsions, simple fracture pattern
	Type III: high-energy injury involving extensive soft-tissue damage or multifragmentary fracture, segmental fractures, or bone loss irrespective of the size of skin wound
AO Muller (9)	A1: Simple spiral fracture
	A2: Simple oblique fracture (>30°)
	A3: Simple Transverse fracture (<30°)
	B1: Spiral wedge fracture
	B2: Bending wedge fracture
	B3: Fragmented wedge fracture
	C1: Spiral complex fracture
	C2: Segmental complex fracture
	C3: Irregular complex fracture
AO Tscherne (8)	1: Minimal skin laceration
	2: Skin laceration, circumferential contusions, moderate contamination
	3: Extensive: major vascular and/or nerve damage, compartment syndrome
	4: Subtotal and complete amputations
OTS (7)	Simple: primary wound closure
	Complex A: wound closure requiring bone shortening or deformation
	Complex B: wound closure requiring soft tissue reconstruction
	Complex C: wound closure requiring vascular repair

AO = Arbeitsgemeinschaft für osteosynthesefragen, and OTS = orthopaedic trauma society.

### Follow-up

Participants were followed up by trained research assistants with face-to-face interviews at 6 weeks, 3 months, 6 months, and 1 year after injury.

### Outcomes

The primary outcome was function, measured by the Short Musculoskeletal Function Assessment (SMFA) dysfunction score at 1 year postinjury (which ranges from 0 [no functional impairment] to 100 [severe functional impairment])^18^ as determined through the SMFA questionnaire, which had been translated into and validated in Chichewa^19^. Complications, such as amputation and signs of fracture-related infection, were assessed at each of the follow-up visits^20^.

### Statistical Analysis

We summarized, using Kruskal-Wallis and χ^2^ tests, the characteristics of participants with different open fracture classifications and compared these between those participants whose fractures were initially managed at the 2 tertiary hospitals and in the 4 district hospitals. The Kendall rank correlation coefficient (τ) was calculated to measure the strength and direction of association between outcome measures and fracture classification^21^. For patient-reported function, this was assessed at each follow-up (6 weeks, 3 months, 6 months and 1 year), whereas for fracture-related infection and amputation, this was at the final follow-up (1 year). Missing data were handled using pairwise deletion, where correlations were computed using all available data for each pair of variables. Observations with missing values in 1 variable were excluded from that specific correlation calculation but retained for other analyses. We interpreted correlation coefficients as negligible (0-0.09), weak (0.1-0.39), moderate (0.4-0.69), strong (0.7—0.89), and very strong (0.9-1.0)^22^. 95% confidence intervals were calculated using bootstrapping, generating 2000 resamples with replacement to estimate the sampling distribution of the correlation coefficients. All analysis used R (version 4.3.1)^23^.

## Results

287 participants were recruited, and 88% (n = 252) photographs and 95% (n = 274) radiographs were included. The median age of participants was 34 years (interquartile range: 26-44), with men comprising 86% (n = 248) of the cohort (Table I). The predominant cause of injury was road traffic incidents 69% (n = 197), primarily involving motorcycles or pedestrians 54% (n = 155).

**Fig. 1:**
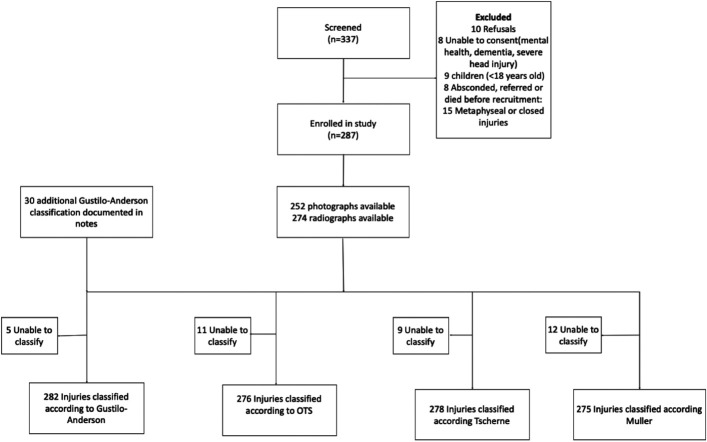
STROBE diagram.

We were unable to classify 6 radiographs (2%) and clinical photographs according to Gustilo-Anderson, 11 (4%) according to OTS, 9 (3%) according to Tscherne, and 12 (4%) according to Muller (Fig. 1). The overall classifications are reported in Table II. Gustilo type III injuries were the most common in tertiary hospitals (107%, n = 107), whereas type I injuries were the most common type of injuries in district hospitals (51%, n = 31) (p-value<0.01). There were more OTS complex injuries that presented to or were referred to tertiary hospitals (14%, n = 26) as compared with district hospitals (4%, n = 2) (p < 0.01). The most common type of AO Muller classification was B3 both in tertiary (22%, n = 42) and district hospitals (21%, n = 10) (p = 0.07). However, there were more type Muller C3 fractures in tertiary hospitals compared with district hospitals (19%, n = 36 vs 8%, n = 4). The most common type of AO Tscherne classification was 2 both in tertiary (66%, n = 124) and district hospitals (52%, n = 25), but there were more type 3 injuries in tertiary hospitals (9%, n = 17) compared with district hospitals (2%, n = 1) (P-value = 0.01).

**TABLE II:** Classification of Open Fractures by Hospital Setting [Missing: 6 (2%) Gustilo-Anderson, 11 (4%) OTS, 9 (3%) Tscherne, and 12 (4%) Muller]

	Tertiary n 224 (%)	Districts n 63	Overall n = 287	p
Median age (IQR), years	34 (26-44)	36 (25-49)	34 (26-45)	0.55
Male	198 (88%)	50 (79%)	248 (86%)	0.10
Gustilo (2% missing, n = 5)				<0.01
Type I	45 (20)	31 (51)	76 (26)	
Type II	69 (31)	18 (30)	87 (30)	
Type III	107 (47)	12 (20)	119 (41)	
AO Muller (4%, n = 12)				0.07
A1	12 (6)	0	16 (6)	
A2	28 (15)	18 (38)	55 (20)	
A3	32 (17)	6 (12)	42 (15)	
B2	31 (16)	9 (19)	47 (17)	
B3	42 (22)	10 (21)	60 (20)	
C2	7 (4)	2 (1)	12 (4)	
C3	36 (19)	4 (8)	46 (17)	
AO Tscherne (18%, n = 53)				0.01
1	43 (23)	20 (42)	72 (26)	
2	124 (66)	25 (52)	180 (65)	
3	17 (9)	1 (2)	20 (7)	
4	5 (3)	2 (4)	7 (3)	
OTS				<0.01
Simple	153 (81)	46 (96)	230 (82)	
Complex A	10 (5)	0	20 (7)	
Complex B	26 (14)	2 (4)	29 (10)	
No. of amputations	16 (7)	2 (3)	18 (6)	<0.01
No. of fracture related infections	71 (31)	10 (6)	77 (27)	<0.01

AO = Arbeitsgemeinschaft für osteosynthesefragen, and OTS = orthopaedic trauma society.

**Fig. 2:**
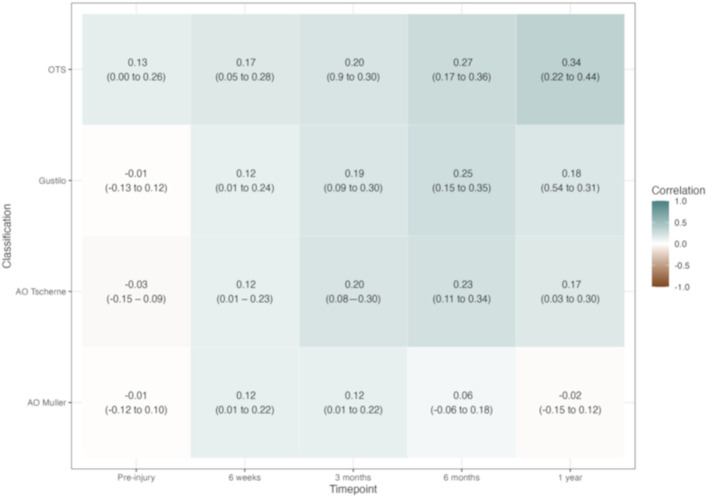
Kendall correlation between SMFA at different time points and classifications [Missing: 6 (2%) Gustilo-Anderson, 11 (4%) OTS, 9 (3%) Tscherne and 12 (4%) Muller]. Timing is after date of injury. AO = Arbeitsgemeinschaft für osteosynthesefragen, and OTS = orthopaedic trauma society.

All the Kendall correlation for open fracture classifications and patient function using SMFA were negligible or weak at all time points post-injury. The Kendall correlation for all classifications except AO Muller improved at 1 year compared with baseline. For Gustilo and AO Tscherne Kendall correlation was highest at 6 months (0.25 (95% confidence interval [CI]: 0.15-0.35) and 0.23 (95% CI: 0.11-0.34), respectively). For OTS, the Kendall correlation was highest at 1 year after injury and was 0.34 (95% CI: 0.22-0.44)—Fig. 2. The Kendall correlation with infection was weak or negligible for all classification and was highest for OTS classification (0.34, 95% CI: 014-0.52). The Kendall correlation with amputation was weak for all classifications and was highest for OTS (0.39, 95% CI: 0.14-0.58)—Table III.

**TABLE III:** Kendall Correlation Between Infection and Amputation 1 Year After Injury And Different Classifications of Injury at Baseline [Missing: 6 (2%) Gustilo-Anderson, 11 (4%) OTS, 9 (3%) Tscherne, and 12 (4%) Muller]

Classification	Infection (95% CI)	Amputation (95% CIs)
OTS	0.34 (0.14-0.52)	0.39 (0.14-0.58)
Gustilo	0.31 (0.17-0.43)	0.38 (0.14-0.59)
AO Muller	0.12 (-0.05-0.26)	0.19 (0.1-0.32)
AO Tscherne	0.24 (0.09-0.38)	0.24 (0.24-0.50)

AO = Arbeitsgemeinschaft für osteosynthesefragen, Ci = confidence interval, and OTS = orthopaedic trauma society.

## Discussion

The main finding of this study is that the current major classifications had weak or negligible correlation with patient-reported function, amputation, and infection over 1 year in a LIC. More research needs to be conducted to develop new or adapt current classifications to improve management of open fracture in LMICs.

The Orthopaedic Trauma Society and Gustilo-Anderson classification has better correlation with patient function at 1 year compared with the Tscherne and Muller classification. This may be due to the Muller classification based on fracture pattern and the Tscherne classification based on soft tissue injuries, whereas OTS and Gustilo are classified based on bone fracture and soft tissue. Indeed, some people advise using a combination of Muller and Tscherne which may improve the correlation with patient function, infection, and amputation^24^. The Muller classification, which is predominantly based on fracture pattern, had the weakest correlation which may suggest the importance of the soft tissue injury in terms of patient function, infection, and amputation over fracture pattern^25^. Any development or adaptation of open fracture classifications for low-income countries should include soft-tissue injuries and reconstructive options available in LMICs.

There was higher proportion of severe injuries compared with other studies. In Malawi, 45% of injuries were classed as Gustilo III injuries compared with 37.4% in a prospective observational study of 31,255 patients in 18 LMICs^26^. This could be due to the difference of populations from these 2 studies with the study by Pouramin et al. being mostly from China and India rather than a LIC. Furthermore, more participants' injuries were caused by road traffic injuries in Malawi as compared with the global collaborative study (68% vs 44%).

According to the OTS classification, there were more simple fractures in Malawi (82%) compared with a prospective cohort study of 1,175 patients with open fractures in the United Kingdom (71%), but this may reflect the lack of open fracture reconstructive options in LICs rather than severity of open fractures. The OTS classification is based on specific treatments for severe open fractures (such as bone deformation, soft tissue coverage, and vascular repair), and the authors acknowledge that the classification might be adapted to the major trauma network system in the United Kingdom. Indeed, a lot of these treatments might not be available in LMICs due to the lack of plastic and vascular resources such as microscopes for surgery^27^. Open fracture classification that is based on treatments should include universal treatment rather than treatments that are mostly available in HICs. Alternatively, orthopaedic capacity needs to be developed to ensure that effective treatments used in open fracture classifications are available across LMICS.

All the current open fracture classifications had weak or negligible association with function, despite the OTS classification showing significant difference in disability and quality of life at 1 year in HICs between simple and complex injuries^6^. This might be due to differences in patient reported outcome measures (PROMs) used, cultural difference, and patient or injuries characteristics^28^. The current classification also had weak or negligible association with infection and amputation, despite the Gustilo classification being associated with these 2 outcomes in HICs^29^. As classifications are used to characterize severity, it is important that classifications are associated with PROMs that are locally relevant and important to patients and clinicians^16^. There are still issues of objectivity and patient-centered outcomes for open fracture classifications in LICs. Enhancing the relevance and applicability of open fracture classifications in LICs necessitates a more nuanced understanding of the local healthcare context and patient needs. This could involve the development of new or adapted classification systems that better capture the complexities of fractures in diverse settings. Currently the Gustilo-Anderson is the most common classification used globally including in Malawi which allows for comparison^10^, and if a new open fracture classification were to be developed in LICs, a lot of training would be required for these classifications to be used by clinicians and researchers locally.

There were several limitations to this study. Variation in treatment of patients with open tibia fractures including timing of debridement, method of fixation, and antibiotic use have previous been shown to influence patient-reported outcomes in low-income countries^2^. However, evaluating the effect of specific treatments was beyond the scope of our study and too few patients received each specific treatment within each fracture classification to allow meaningful adjustment for treatment as a covariate. These variables are common in low-resource settings, and their presence highlights the need for a classification system that remains applicable under such conditions. Future analyses incorporating these factors may provide further insights into their specific impact on outcomes. Our findings also reinforce the importance of considering the trauma system as a whole when assessing outcomes^30^. Although most patients in our cohort underwent debridement within a median of 1 day of injury, delays to definitive fixation were more variable delays are associated with increased risk of fracture-related infection^2^. Future analyses examining outcomes by delay to tertiary care, particularly among patients with severe open fractures, may help inform referral strategies and optimize care delivery across the system.

Many other classification scores have been described for open fractures; however, these often focus only on the severe end of the injury spectrum such as the Mangled Extremity Severity Score^31^ or require detailed information that was not available in this study such as the Ganga Hospital Open Injury Score or Orthopaedic Trauma Association classification^32,33^. Although injury mechanism, contamination type, and comorbidities are recognized as important confounders, they were either infrequent or inconsistently recorded in this cohort. The majority of injuries resulted from high-velocity road traffic crashes, whereas farm injuries and organic contamination were rare^2^. Only a small proportion of patients had documented comorbidities, which may reflect limited screening rather than true absence^2^. These limitations restrict our ability to analyze the impact of these factors in detail.

Interobserver variability has been reported for Gustilo-Anderson classification, which could lead to misclassification bias and mismanagement of open fractures^34,35^. Study reviewers were not blinded to initial field classifications, which may have introduced bias introduced by prior assessments. Radiographs and clinical photographs were not available for all patients; in some cases, such as those with severely mangled limbs, radiographs were not obtained, and in others, equipment failures (e.g. nonfunctional radiograph machines) contributed to missing imaging, limiting the ability to apply classification systems consistently. Any development of open fracture classifications in LICs should be assessed for interobserver and intraobserver variation to assure objectivity, reproducibility, and consistency. Furthermore, the Gustilo-Anderson classification should occur at the time of initial debridement and the preoperative photographs might not be the same as intraoperative clinical classification.

The research assistants were trained to be consistent with administering locally validated questionnaires^19^, but the study relies on patient-reported outcomes for function, which may introduce bias or inaccuracies due to self-reporting^36^. The SMFA has been validated and used in Malawi for various types of injuries, allowing for comparison across studies^37^. It assesses important domains such as mobility, daily activities, emotional well-being, and social function. Local qualitative research supports the relevance of these domains to patients’ experiences^16^. However, no single tool can capture all aspects of function, and the SMFA may miss locally important activities such as farming or walking long distances. As a result, some relevant outcomes may not have been fully reflected in our findings. Therefore, more mixed methods studies could be conducted to gain deeper insights into patient experiences and outcomes, thereby complementing the numerical data with context-rich information.

The study's findings are based on data from 6 hospitals in Malawi, which may not be generalizable to other LICs with different healthcare systems, patient populations, or resources. Future studies could be conducted in other LICs, and open fracture classifications development for LMICs could involve international bodies that would be more representative of multiple LICs.

In conclusion, existing open fracture classification systems, largely developed in high-income settings, show limited correlation with outcomes such as function, infection, and amputation in low-income contexts. This may be because treatment factors and other known confounders, such as delays to care or host factors, play a greater role in determining outcome than classification alone. Nevertheless, to be clinically and scientifically useful, classification systems must account for such variability and remain applicable in real-world settings. There is a need to adapt or develop systems that reflect the realities of low-resource environments and better support decision making and outcome evaluation.
